# Comparative structural studies on Bovine papillomavirus E6 oncoproteins: Novel insights into viral infection and cell transformation from homology modeling and molecular dynamics simulations

**DOI:** 10.1590/1678-4685-GMB-2023-0346

**Published:** 2024-08-09

**Authors:** Lucas Alexandre Barbosa de Oliveira Santos, Tales de Albuquerque Leite Feitosa, Marcus Vinicius de Aragão Batista

**Affiliations:** 1 Universidade Federal de Sergipe, Centro de Ciências Biológicas e da Saúde, Departamento de Biologia, Laboratório de Genética Molecular e Biotecnologia (GMBio), São Cristóvão, SE, Brazil.

**Keywords:** Bovine papillomavirus, E6 oncoprotein, molecular dynamics simulations, CBP/p300, hMCM7.

## Abstract

Bovine papillomavirus (BPV) infects cattle cells worldwide, leading to hyperproliferative lesions and the potential development of cancer, driven by E5, E6, and E7 oncoproteins along with other cofactors. E6 oncoprotein binds experimentally to various proteins, primarily paxillin and MAML1, as well as hMCM7 and CBP/p300. However, the molecular and structural mechanisms underlying BPV-induced malignant transformation remain unclear. Therefore, we have modeled the E6 oncoprotein structure from non-oncogenic BPV-5 and compared them with oncogenic BPV-1 to assess the relationship between structural features and oncogenic potential. Our analysis elucidated crucial structural aspects of E6, highlighting both conserved elements across genotypes and genotype-specific variations potentially implicated in the oncogenic process, particularly concerning primary target interactions. Additionally, we predicted the location of the hMCM7 binding site on the N-terminal of BPV-5 E6. This study enhances our understanding of the structural characteristics of BPV E6 oncoproteins and their interactions with host proteins, clarifying structural differences and similarities between high and low-risk BPVs. This is important to understand better the mechanisms involved in cell transformation in BPV infection, which could be used as a possible target for therapy.

## Introduction

There are 44 Bovine papillomaviruses (BPV) classified into five genera so far, being the high-risk type BPV-1 a member of the *Deltapapillomavirus* genus, and the low-risk type BPV-5 a member of the *Epsilonpapillomavirus* genus (PaVE, 2023) (Daudt et al., 2018). The BPV-1 pathology is characterized by the benign formation of hyperplasic lesions of both cutaneous and mucosal tissue that generally regress. Still, it may also persist in the presence of environmental carcinogenic co-factors, leading to cancer. BPV-5 also leads to the benign formation of hyperplasic lesions in the same tissue, but there have been no reports of its persistence to cancer. These two genera share the presence of the E6 oncogene and other genetic characteristics, making it particularly interesting for evolutionary studies (Campo, 2006; Van Doorslaer, 2013).

BPV E6 oncoproteins are ~135 amino acids long, and composed of two zinc-binding domains with a linker helix domain in between, which forms a hydrophobic pocket (Zanier et al., 2013). The E6 protein binds to the paxillin LD1 motif (MDDLDALLAD) via a network of residues forming a basic-hydrophobic pocket on its surface (Tong and Howley, 1997; Tong *et al.*, 1998; Zanier *et al.*, 2013). The selection of residues at the C and N terminus of the peptide may be influenced by the nature of the residues from the N-terminus of the E6 N-domain and the C-terminus of its linker helix, respectively (Zanier *et al.*, 2013). BPV-1 E6 oncoprotein has been reported to interact primarily with Mastermind-like protein 1 (MAML1), MAML3, E3 ubiquitin ligase (E6AP/UBE3A), signal transduction adaptor protein paxillin, transcription factor activator protein 1 (AP1), and calcium-binding protein E6-BP/ERC-55, through its hydrophobic pocket (Chen et al., 1995, 1998; Tong and Howley, 1997; Tong *et al.*, 1998; Das et al., 2000; Brimer et al., 2012; Tan et al., 2012). BPV-1 E6 has also been reported to bind secondarily to the coactivator family CBP/p300 (Zimmermann et al., 2000) and human minichromosome maintenance protein hMCM7 through the N-terminal of the E6 surface (Kukimoto et al., 1998). Other interacting protein partners may yet to be discovered. 

E6 self-association propensity and strong interaction with LXXLL motifs suggest that these molecules preferentially exist in a complex condition in the host cell and it is likely to adopt a different overall structure without the bonded peptide (Zanier et al., 2012). Competitively charged leucine peptide can repress murine C127 cell transformation by BPV-1 E6 *in vivo* (Bohl et al., 2000), demonstrating the importance of this interaction for transformation. It has also been suggested that multiple interactions by E6 with LXXLL motifs with other host proteins are required for malignant transformation (Wade et al., 2008; Vande Pol and Klingelhultz, 2013).

CBP/p300 regulates the transcription of genes via chromatin remodeling. It interacts secondarily with BPV-1 E6 in the same manner as it does with HPV-16 E6, through its C-terminus domain, resulting in the inhibition of CBP/p300, which results in the downregulation of p53 transcription. This binding site has lower conservation between BPV-1 and HPV-16 E6 than it has between BPV-1 and BPV-5 E6. However, it is not known whether low-risk BPV E6 makes this interaction. Although this interaction is necessary, it is not sufficient for host cell transformation (Zimmermann et al., 2000; Thomas and Chiang, 2005).

BPV-1 E6 also binds secondarily to hMCM7, a key component of the cellular pre-replication complex (Zimmermann et al., 2000). This interaction has been mapped to the N-terminus of HPV-16 E6 (Kukimoto et al., 1998). Yeast two-hybrid experiments show a stronger binding of high-risk HPVs E6 to hMCM7 than low-risk HPVs. BPV-1 E6 is less oncogenic than HPV-16 E6 and also binds weaker to hMCM7 (Kukimoto *et al.,* 1998; Zimmermann *et al.,* 2000). It has also been speculated that BPV E6 interacts with secondary associated proteins through its first 10 amino acids, all of which can be deleted without abolishing E6 ability to bind to LXXLL motifs (Meyers et al., 1992; Vande Pol and Klingelhultz, 2013; Zanier et al., 2013).

These oncoproteins have been difficult to study due to their problems in forming crystals with enough quality for their crystallographic structural resolution. Therefore, modeling through computational approaches has been used in these situations enabling the exploration of the protein structure (Baker and Sali, 2001; Tunyasuvunakool et al., 2021; Akdel et al., 2022). Then, this study aimed to perform comparative structural analyses with the E6 oncoprotein of low-risk BPV-5 and high-risk BPV-1 and their interaction with host proteins, which could serve as the basis to better understand the mechanisms involved in cell transformation during BPV infection.

## Material and Methods

### Sequence retrieval and template selection

The amino acid reference sequence of the BPV-5 E6 oncoprotein (accession NP_694430.1) was acquired from the NCBI protein database (NCBI, 2023). 

A blast search was carried out with the E6 amino acid sequence from BPV-5 against the Protein Data Bank (PDB) using Blast+ 2.2.26 (blastp algorithm) for template selection (Camacho et al., 2009). The selected template was the E6 of BPV-1 (PDB accession number: 3PY7), which presented an E-value of 9e-21, 38% of sequence identity, 51% of similarity, and 92% of sequence coverage. The template crystal structure was downloaded from PDB (2023). Due to its large flexibility, the first 10 and the last 7 amino acids of the template are absent in the crystal structure, so for these regions, only its amino acid sequence was considered for comparisons to BPV-5 E6 protein in this study.

### Sequence alignment and comparison

BPV-1 E6 and BPV-5 E6 amino acid sequences were aligned using MEGA6 software (Tamura et al., 2013). The E6 protein was divided into three domains of 48, 14, and 72 amino acids, defined as the N-terminal zinc-binding domain, the linker helix, and the C-terminal zinc-binding domain, respectively. The sequences were analyzed and their domains were compared in terms of conservation, similarities, and differences.

### Homology modeling of BPV-5 E6 oncoprotein

The LXXLL LD1 binding motif of paxillin was maintained in the template sequence for the homology modeling of BPV-5 E6 protein. This feature improves the quality of the model by constructing the structure closer to its native conformation. For this reason, the paxillin found in the template was also added to the query sequence of the BPV-5 E6 protein after a docking confirmation. Modeller 9.10 program was used for the homology modeling, which aligns the sequence to be modeled with a known related structure, implementing satisfaction of spatial restraints and *de novo* modeling of loops (Sali and Blundell, 1993; Fiser and Sali 2003; Webb and Sali, 2016).

### Quality assessment and structure refinement

First, the models had their structure assessed using the Ramachandran plot in Procheck 3.4 (Laskowski et al., 1993). The highest-ranked models in terms of quality assessment values were then refined using the ModRefiner server for high-resolution protein structure refinement (Xu and Zhang, 2011). ModRefiner refines proteins by performing a two-step atomic-level minimization of its Cα traces, addressing the unphysical local distortions issue. With the use of a composite of physics and knowledge-based force field, the side chain rotameters and backbone atoms were refined (Xu and Zhang, 2011). These structures were all assessed again based on the Ramachandran plot after refinement. Furthermore, visual inspections were carried out throughout the process.

### Energy minimization and molecular dynamics

To assess the stability of the models and perform an associated conjugate gradient energy minimization, molecular dynamics simulations with the refined models and the template were performed using NAMD 2.9 (Phillips et al., 2005), which were done with and without the LD1 paxillin and the MAML1 protein ligands. These ligands were docked with the use of ClusterPro and ZDOCK servers (Kozakov et al., 2013; Pierce et al., 2014). All simulations were run for 15 nanoseconds (ns), with 2000 steps of conjugate gradient minimization with no restraints, 0.5 mol/L NaCl ions were added to neutralize the solution, CHARMM22 force field with CMAP corrections to improve backbone behavior, and the water model adopted in this force field was the 3-site TIP3P. The x, y, and z values for the water box dimensions were 72, 54, and 54, respectively. Hydrogen atoms were added to the proteins using psfgen.exe available in VMD 1.9.3 (Humphrey et al., 1996). The temperature rises after minimization from step 2000 to step 11400, from 166 K to 310 K and maintains this temperature until the end of the simulation. The simulations were performed in an Intel Xeon CPU E5-1620 v3 workstation with 8 processors.

### Structural comparison analysis between BPV-1 and BPV-5 E6 oncoproteins

BPV E6 oncoproteins have many structural aspects to be considered for comparison, among the most significant are the residues participating in the binding with the LXXLL paxillin motif, the C and N-terminal zinc-binding domains, the linker helix, the first 10 amino acids of the N-terminal domain, the structural aspects of the hydrophobic pocket, the residues nature located at the N-terminus of E6 N-domain, the C-terminus of its linker helix, the CBP/p300 binding domain and the putative hMCM7 binding site.

To quantitatively and qualitatively compare these aspects between the E6 protein of BPV-1 and BPV-5, VMD 1.9.3 (Humphrey et al., 1996) and Chimera 1.11 (Pettersen et al., 2004) were used for visualizing and manipulating the molecule, and MultiSeq 2.4 software was used for their structural alignment (Roberts et al., 2006). 

## Results

The Ramachandran plot showed 96.8% of the residues located in the most favored regions and no residues in the generally allowed or disallowed regions (Table 1). Expected values for stereochemical parameters in well-resolved structures have >90% of phi and psi angles in the core region of the plot. The higher the value the more amino acid residues are located in the most energetically favorable regions (Morris et al., 1992). 

**Table 1-:** Quality assessment values of BPV-5 E6 representative structure. Well-solved strutuctures have over 90% Phi/Psy angles in the core region of favorable energy in the Ramachandran plot. *Selected structure.

Structure	Ramachandran			
	Core	Allow	Gener	Disall
B1E6	95.3%	4.7%	.0%	.0%
B1E6 with paxillin LD1	93.0%	7.0%	.0%	.0%
B1E6MAML1	93.9%	6.1%	.0%	.0%
B5E6*	96.8%	3.2%	.0%	.0%
B5E6 with paxillin LD1	94.4%	4.8%	.8%	.0%
B5E6MAML1	96.1%	3.9%	.0%	.0%

**Figure 1.**
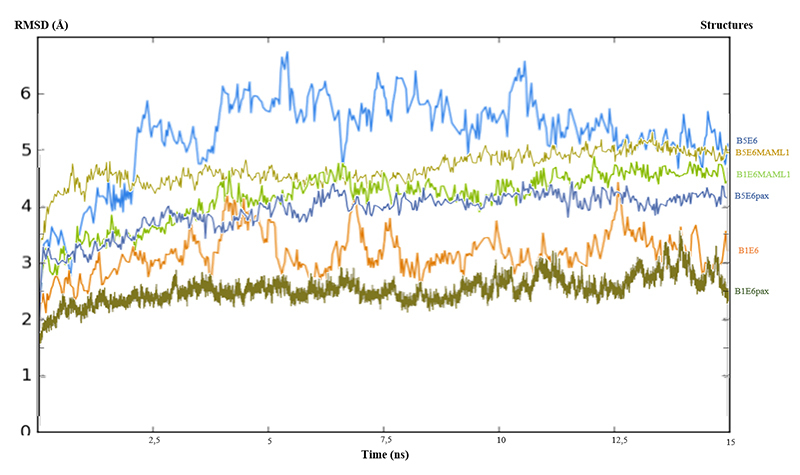
Molecular dynamics simulation demonstrating that all structures are stable in ionized waterbox conditions. Equilibrium simulation were run for 15 ns using NAMD.

The molecular dynamics simulations protocol presented here not only certified the structures’ stability but also corroborates with the suggestion that these molecules have less stability alone than bonded with their cellular ligand (Figure 1) (Zanier et al., 2013). Furthermore, the structure of BPV-5 E6 bound to LXXLL motifs showed greater instability compared to the BPV-1 E6 complexes, suggesting that although BPV-5 E6 can interact with these targets, it is likely that the strength of the interaction is less than that occurring in E6 of BPV1 (Figure 1).

**Figure 2-:**
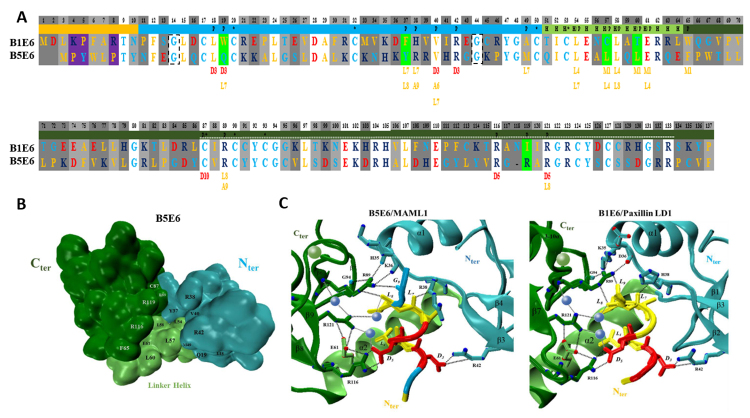
A: Alignment of BPV-1 E6 (top), BPV-5 E6 (bottom), and paxillin residues (two last bottom lines). Hydrophobic residues are labeled in yellow, polar in cyan, acid in red, and basic in dark blue. Predicted secondary interaction (yellow squares). Non-conservative variations of the predicted secondary interaction sequence (purple). N-terminal zinc-binding domain (cyan square). C-terminal zinc-binding domain (dark green square). Linker helix domain (H - lime). Conserved residues (white square). Conservative replacement (light gray square), different residues with similar physicochemical properties. Non-conservative variations (dark gray square). Paxillin binding residues (P). Non-conservative variations of paxillin binding residues (light green square). CBP/p300 binding site sharing a 49% identity (silver dotted line). Conserved cysteines that bind to zinc forming the C and N terminals zinc domains (*). The two glycine residues in the 14 and 44 positions circled with dotted squares are the putative conserved hMCM7 binding residues. B: 3D Representation of the BPV-5 E6 domains. The N-terminal domain is in cyan, the linker helix is in lime, and the C-terminal domain is in dark green. The motif interaction network is highlighted. C: Networks of E6/LXXLL peptide interaction of BPV-5 E6 with MAML1 (left) and BPV-1 E6 with paxillin LD1 (right). The peptide residues and the tips of the oncoprotein interaction residues are colored according to their nature, the zinc atom is represented by a green sphere while water molecules are represented with blue spheres. The N-terminal is colored in cyan, the linker helix in lime, and the C-terminal in dark green. Dashed lines represent the mode of interaction between key residues.

The BPV-1 and BPV-5 E6 sequences have 137 and 134 amino acid residues, respectively. They shared 38% identity and 51% similarity in total, 34% identity for the N-terminal, 50% for the linker helix, and 31% for the C-terminal. The presence of cysteines that bind to zinc, which forms the C and N terminals zinc-binding domains, are very conserved among both E6 oncoproteins. These residues are located at positions 17, 20, 50, 53, 87, 90, 93, 124, and 127 of the alignment, represented as CXXC, being one of the main reasons for the overall fold of the domains (Figure 2 A ).

**Figure 3-:**
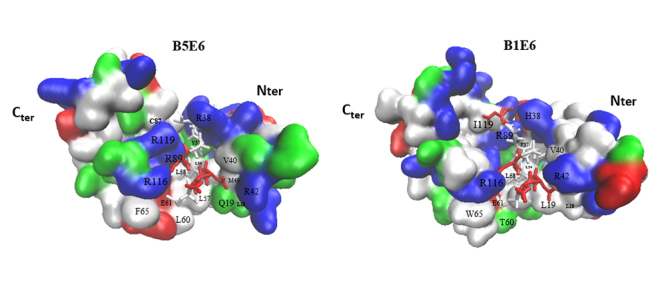
On the left, the BPV-5 E6 protein model, and on the right BPV-1 E6 protein, both bonded to paxillin. Ligand binding residues of BPV-1 E6 were aligned with BPV-5 E6 and labeled here. Residues are colored in respect to their nature, hydrophobic (white), polar (green), basic (blue), and acidic (red).

BPV-5 E6 protein structure has 117 amino acid residues, the first 10 and the last 7 residues are not represented in the three-dimensional structure (Figures 2 B  and 3). Positively charged residues surround the hydrophobic pocket and interact with the acidic residues of the binding motif, which is also seen in the BPV-1 E6 protein (Figure 3). The peptide interacting network is shorter concerning the number of residues, with 18 ligand-binding residues, whereas BPV-1 E6 has 20. Of these, 10 residues are identical, three are similar alterations, and five are dissimilar alterations (Figures 2 C  and 3). The N-terminal and linker helix domains together have four peptide binding residue variations, while the C-terminus has only one. Therefore, there is a possibility that the N-terminal and linker helix domains may be the main domains in the divergence of interactions with other proteins, such as paxilin and MAML1, which are the more conserved domains between BPV-1 and BPV-5 E6 proteins.

**Figure 4-:**
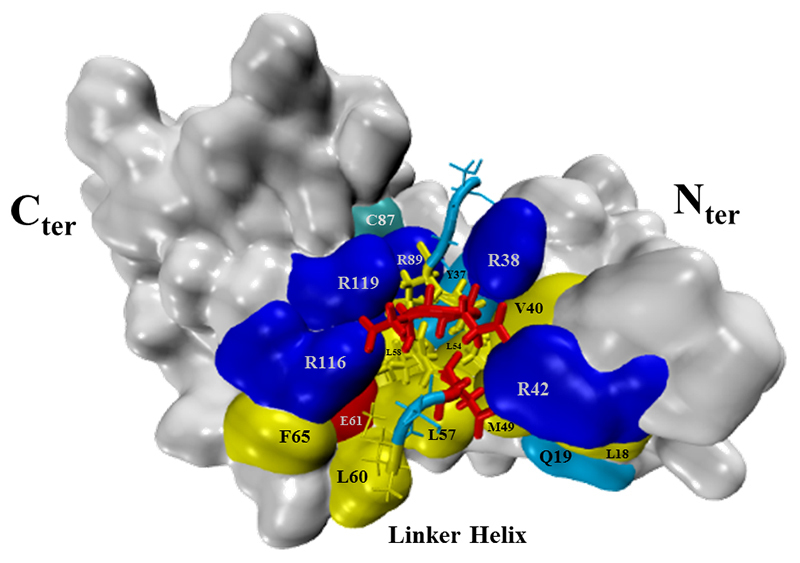
BPV-5 E6 with MAML1 10 amino acid sequence containing the LXXLL motif docked to its basic-hydrophobic cavity.

The majority of the MAML1-contacting residues from the cavity of BPV-5 E6 seem to have preserved very similar contact features as in BPV-1 E6. Some exceptions are amino acids Q19 and L18 from the N-terminus domain, which is not in contact with the MAML1 peptide, as occurred in BPV-1 E6 with W19 and L18, and residues R119 and Y37 which have different physicochemical properties (Figure 4). Less residues participating in the interaction network could indicate less variety of possible interactions and therefore possibly limit the number of different LXXLL motifs it can bind to, and the residue variation could alter the mode of interaction with the peptide.

The first 10 and 8 amino acids of the N-terminal of BPV-1 and BPV-5 E6, respectively, which are known to be flexible, have 10% identity, 40% similarity, three amino acid substitutions, and two indels, which represent 50% of this region. Upon examining the last seven residues, it is evident that two are conserved while five exhibit variation across the genotypes.

## Discussion

BPV is of great veterinary and evolutionary interest. However, despite their importance and high diversity, there are only a few available 3D structures of BPV proteins due to experimental difficulties, and so studies that consider such structural aspects, although important, are scarce. Therefore, this study has taken advantage of the bioinformatics approach to bring novel insights into the structural and functional features of BPV E6 oncoprotein.

BPV-5 E6, as in its homologous BPV-1 E6, presents a basic-hydrophobic cavity surrounded by positively charged residues favoring the interaction with the acidic moieties of the peptide (Figures 2 C  and 3). R^116^ seems to have conserved the same strategic position as in BPV-1 E6, also interacting with acidic residues E^61^ from the linker helix and D^5^ of the MAML1 peptide. The same is true for the conserved positions of R^89^ and R^121^, though R^89^ interacts with residue H^35^ and residue K^36^ of the N-terminal domain. From this perspective, this data suggests that R^116^, R^89,^ and R^121^ from BPV-5 E6 are playing important roles in its structural maintenance as it is for BPV-1 E6. D^3^ interacts with the conserved residue R^42^ and L^7^ with residue R^38^, both seem to be interacting similarly as for BPV-1 E6. Curiously, its N-terminal and linker helix domains together have four non-conserved peptide binding residue differences while the C-terminus only has one. This could suggest that the N-terminal and linker helix domains may be the main influential domains in the divergence of peptide interaction between BPV-5 and BPV-1 E6s. The majority of the hydrophobic peptide-contacting residues from the cavity seem to have conserved very similar contact features as in BPV-1 E6, with the exceptions of Q^19^ and L^18^ from the N-terminus domain, which are not in contact with the peptide as their BPV-1 E6 counterparts W^19^ and L^18^, respectively, and residues R^119^ and Y^37^ which are non-conserved variations (Figures 2 C  and 3). 

Less residues participating in the interaction network could indicate less variety of possible interactions and therefore possibly limit the number of different LXXLL motifs it can bind to, and the non-conserved differences could be altering the mode of interaction with the peptide. The simulations of the BPV-5 E6 complexes presented higher RMSD values than the BPV1 E6 complexes, which suggests that the strength and stability of the interaction of the non-oncogenic genotype is lower than that of the oncogenic genotype for the LXXLL motifs evaluated (Figure 1). The nature of E6 putative peptide selecting sites located at its linker helix C-terminal and N-terminal of the N-domain are hydrophobic and basic-polar respectively, as for BPV-1 E6 is polar-hydrophobic and basic. We performed a simulation equilibration attempt with a well-validated model linked to paxillin with no success, though the simulation of the same model alone was able to equilibrate, suggesting that this molecule had greater stability alone than with the paxillin LD1 motif. Interestingly, BPV-5 E6 was modeled and simulated for equilibration bonded to the MAML1 LXXLL motif with success. It is reasonable to speculate that BPV-5 E6 does not bind to paxillin as the data may suggest, since paxillin functions through membrane contacts that seem to influence cell shape (Zimmermann et al., 2000). In this sense, the present protocol should be able to help in the evaluation of E6s’ discriminative interactions with different LXXLL motifs.

The current explanation for the main difference between low and high-risk E7 is that differences in their LXCXE motifs make high-risk E7 bind and target for degradation an extra partner that results in the stabilization of p53 suppressor protein (Zhang et al., 2006). Similarly, E6 oncoproteins can also be evolving in a way by favoring a higher oncogenic potency to proteins with additional and influential binding partners regarding less oncogenic E6 proteins. Therefore, E6’s ability to bind to a bigger variety of LXXLL motifs could raise the possibility of interacting with a more influential partner and in that way becoming more oncogenic. This corroborates the suggestion that multiple interactions with LXXLL motifs on multiple partners may be required for full transformation (Vande Pol and Klingelhultz, 2013). In addition, phylogenetic evidence indicates that E6 and E7 evolved from a common ancestral single-domain protein similar to E6 present in avian papillomaviruses (Van Doorslaer, 2013). If this is the case, then the chances of E6 and E7 sharing very similar evolutionary mechanisms are higher and E6 may be possibly evolving similarly to E7, that is, through the acquisition of a larger variety of binding partners and eventually more influential proteins for stabilizing the host cell transformation.

Secondary interaction with CBP/p300 has been conserved between HPV-16 E6 and BPV-1 E6 on its C-terminus domain, and both bind in the same way (Zimmermann et al., 2000; Thomas and Chiang, 2005). This binding site between BPV-1 and HPV-16 E6s shares 32% sequence identity while it shares 49% between BPV-1 and BPV-5. This can indicate that, since the same mode of interaction is conserved between sequences holding a smaller identity value, then this same mode of interaction to CBP/p300 may happen for BPV-5 E6 as well since it is evolutionary closer to BPV-1. If so, BPV-5 E6 should be able to reduce p53 transcription by binding to CBP/p300. Although this interaction is necessary, it is not sufficient for host cell transformation (Zimmermann *et al.*, 2000), and BPV-5 E6 is reported to be only benign, corroborating this possibility. 

BPV-1 E6 also binds secondarily to hMCM7, a key component of the cellular pre-replication complex. This interaction has been mapped to the N-terminus of HPV-16 E6 (Kukimoto et al., 1998). Yeast two-hybrid experiments show a stronger binding of high-risk HPVs E6 to hMCM7 than low-risk HPVs, BPV-1 E6 is less oncogenic than HPV-16 E6 and also binds weaker to hMCM7 (Kukimoto *et al.*, 1998; Zimmermann et al., 2000). Since the first 10 amino acids from BPV-1 E6 can be absent for it to still be capable of binding to LXXLL motifs, these extra residues have been suggested to enable secondary interactions (Zanier et al., 2013). Curiously, high-risk PVs have these extra sequences ranging from 23-25 residues (Vande Pol and Klingelhultz, 2013; Zanier *et al.*, 2013). When BPV-5 and BPV-1 E6s sequences are aligned, BPV-5 presents 8 residues aligned to these first 10 residues from BPV-1 E6, and no charged residues compared to 3 charged residues in BPV-1 E6. One could speculate what type of effect this shortening of residues and lesser amount of charged residues have on the protein interaction ability, and if it has any connection to the hMCM7 interaction site. Although HPV-16 E6 oncogenicity seems to be independent of hMCM7 binding, it still could be influencing the transformation mechanism (Zimmermann *et al*., 2000).

Multiple studies have demonstrated that E6 proteins from high-risk HPV genotypes interact with cellular proteins containing the PDZ domain through the PDZ-binding motif (PBM), located in the C-terminal region (Ganti et al., 2015; Thomas et al., 2016; Lee et al., 2022). E6 sequences from bovine and other ruminant PVs lack the PDZ-binding motif (ETQL) (Daudt et al., 2018), suggesting that they do not interact with such targets. Indeed, to date, no study has evaluated the interaction between BPV E6 proteins and PDZ targets. The findings of this study indicate that such an interaction does not occur.

This work has added knowledge on the molecular interactions of BPV-5 E6, which could help to elucidate why some BPVs are related to cancer and others are not, providing a potential avenue for therapeutic targeting. However, *in silico* comparative studies, while crucial for understanding structural aspects, have limitations. Novel studies using complementary approaches (Rao et al., 2014) are necessary to further understand the contribution of each residue to the functional and structural interactions of E6 with its targets and how this varies between different genotypes.
